# Supplementary material for: Cryo-EM analysis of the HCoV-229E spike glycoprotein reveals dynamic prefusion conformational changes
Source: Nat Commun. 2021 Jan 8;12:141. doi: 10.1038/s41467-020-20401-y (PMC7794242; doi:10.1038/s41467-020-20401-y)
Supplement: Supplementary file 1 — Supplementary Information [file 41467_2020_20401_MOESM1_ESM.pdf]

Supplementary Information

Cryo-EM analysis of the HCoV-229E spike glycoprotein reveals dynamic prefusion conformational changes

Xiyong Song[1,2,5,#], Yuejun Shi[1,3,#], Wei Ding[6,#], Tongxin Niu[7], Limeng Sun[1,3], Yubei Tan[1,3], Yong Chen[2,5], Jiale Shi[1,3], Qiqi Xiong[1,3], Xiaojun Huang[7], Shaobo Xiao[1,3], Yanping Zhu[2], Chongyun Cheng[2], Zhen F. Fu[1,3,4], Zhi-Jie Liu[8,9,*], Guiqing Peng[1,3,*]

[1] State Key Laboratory of Agricultural Microbiology, College of Veterinary Medicine, Huazhong Agricultural University, Wuhan, China
[2] National Laboratory of Biomacromolecules, Institute of Biophysics, Chinese Academy of Sciences, Beijing, China
[3] Key Laboratory of Preventive Veterinary Medicine in Hubei Province, The Cooperative Innovation Center for Sustainable Pig Production, Huazhong Agricultural University, Wuhan, China
[4] Departments of Pathology, College of Veterinary Medicine, University of Georgia, Athens, GA, USA
[5] University of Chinese Academy of Sciences, Beijing, China
[6] CAS Key Laboratory of Soft Matter Physics, Institute of Physics, Chinese Academy of Sciences, P.O.Box 603, Beijing, China
[7] Center for Biological Imaging, Institute of Biophysics, Chinese Academy of Sciences, Beijing, China
[8] Institute of Molecular and Clinical Medicine, Kunming Medical University, Kunming, China
[9] iHuman Institute, ShanghaiTech University, Shanghai, China

[#] These authors contributed equally to this work

[*] Correspondence to Zhi-Jie Liu (liuzhj@shanghaitech.edu.cn) and Guiqing Peng (penggq@mail.hzau.edu.cn)

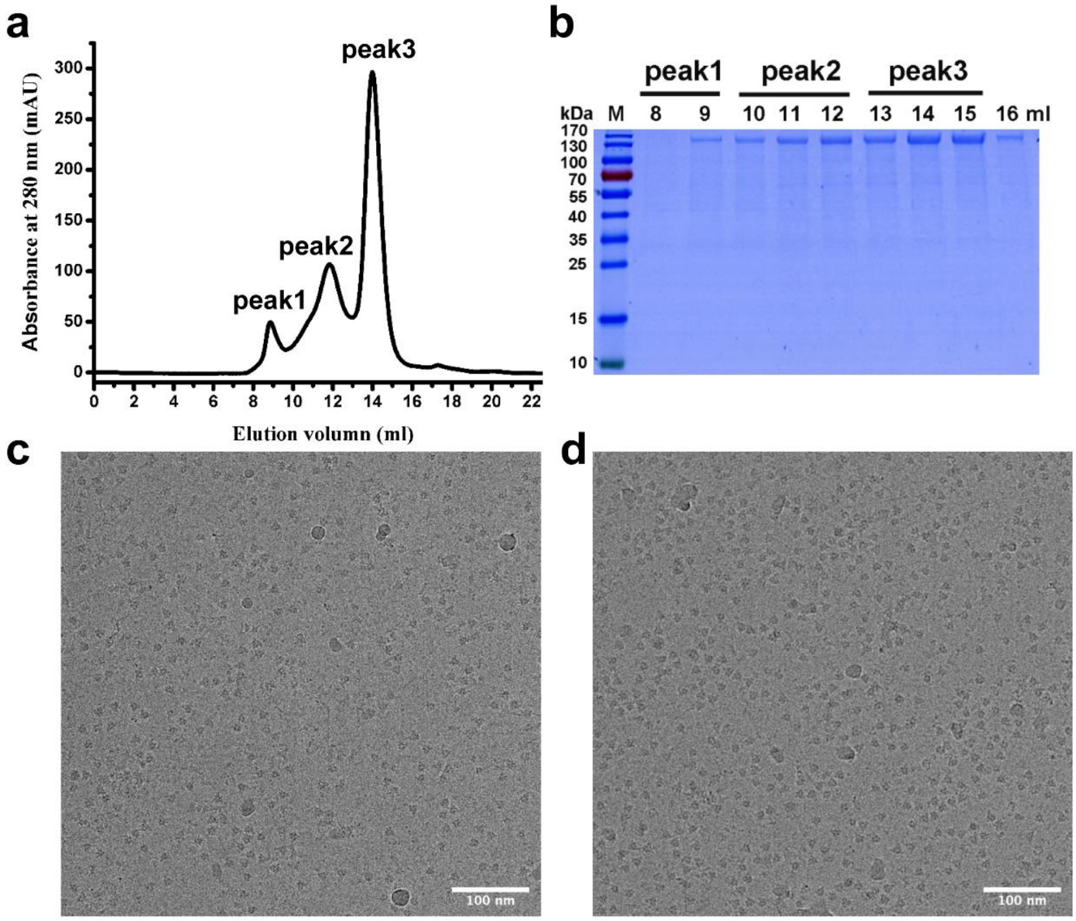

Supplementary Figure 1. Protein characterization of the HCoV-229E S trimer.

(a) Gel filtration profile of the HCoV-229E S trimer. Three peaks are recognized in the gel filtration curve, termed peak 1, peak 2 and peak 3, respectively. (b) SDS-PAGE analysis of the HCoV-229E S trimer. The samples from different elution volumes are labeled as described in panel A. (c) Representative cryo-EM raw micrograph of conformation 1 (derived from peak 2) is shown. (d) Representative cryo-EM raw micrograph of conformation 2 (derived from peak 3) is shown. These data in the Supplementary Figure 1b-d were repeated 3 times independently with similar results.

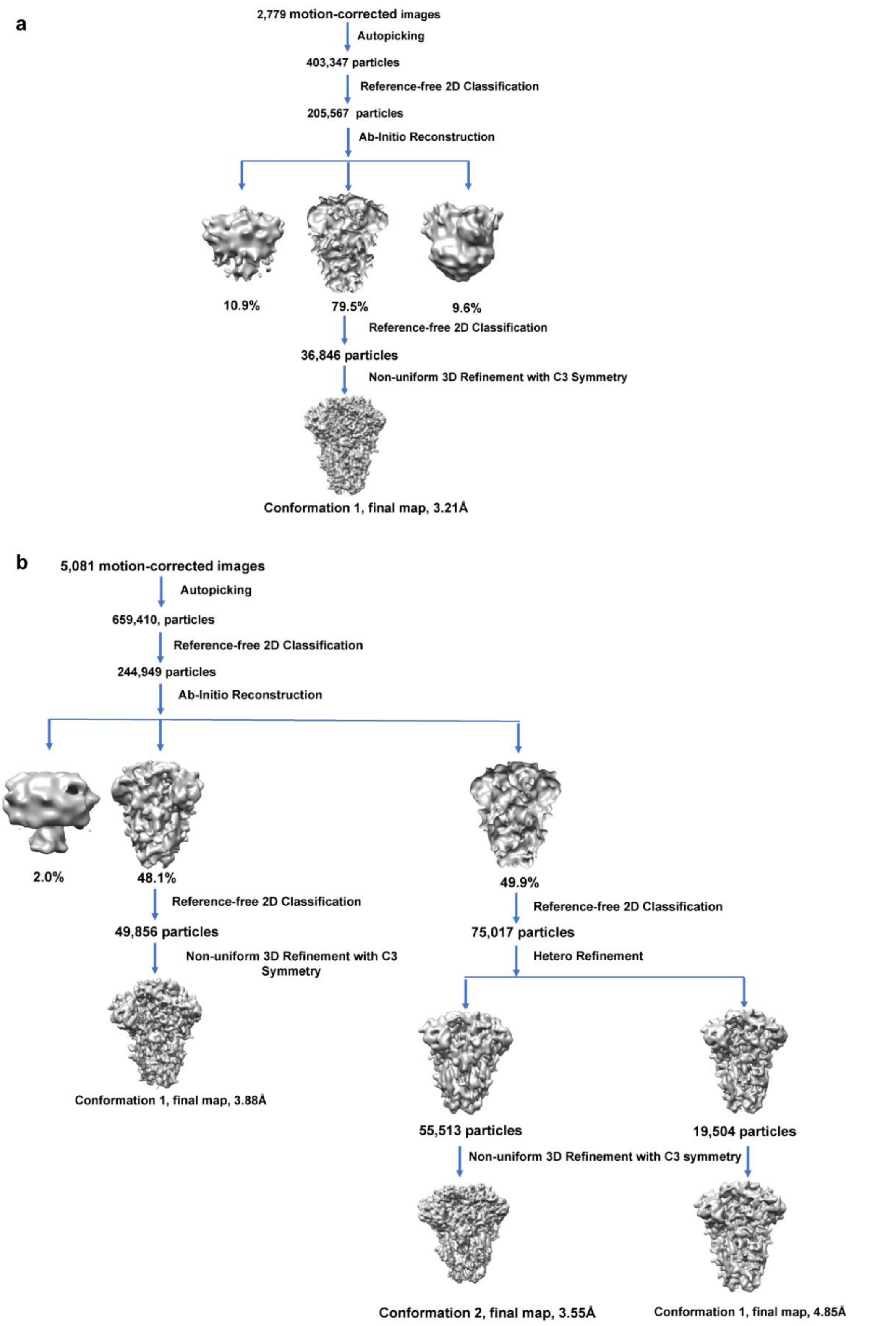

Supplementary Figure 2. Flowchart for cryo-EM data processing of peak2 (a) and peak3 sample (b)

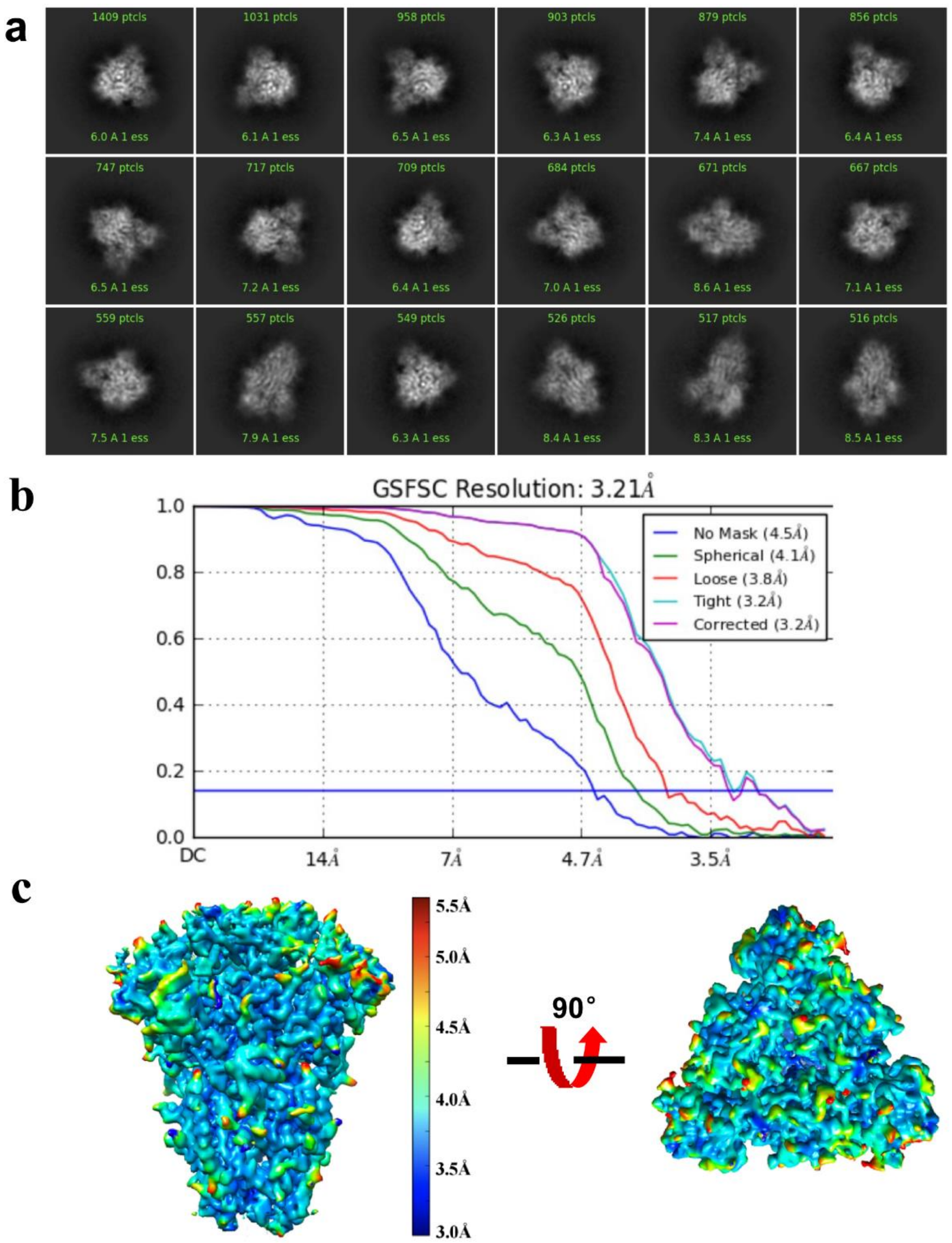

Supplementary Figure 3. Cryo-EM analysis of the conformation 1.

(a) Representative 2D class averages of the conformation 1 used for the final refinement. (b) Gold-standard Fourier shell correlation (FSC) curves of conformation 1. The 0.143 cut-off value is indicated by a horizontal cyan line. (c) Local resolution map of conformation 1 density map analyzed by the local resolution estimation tool in cryoSPARC.

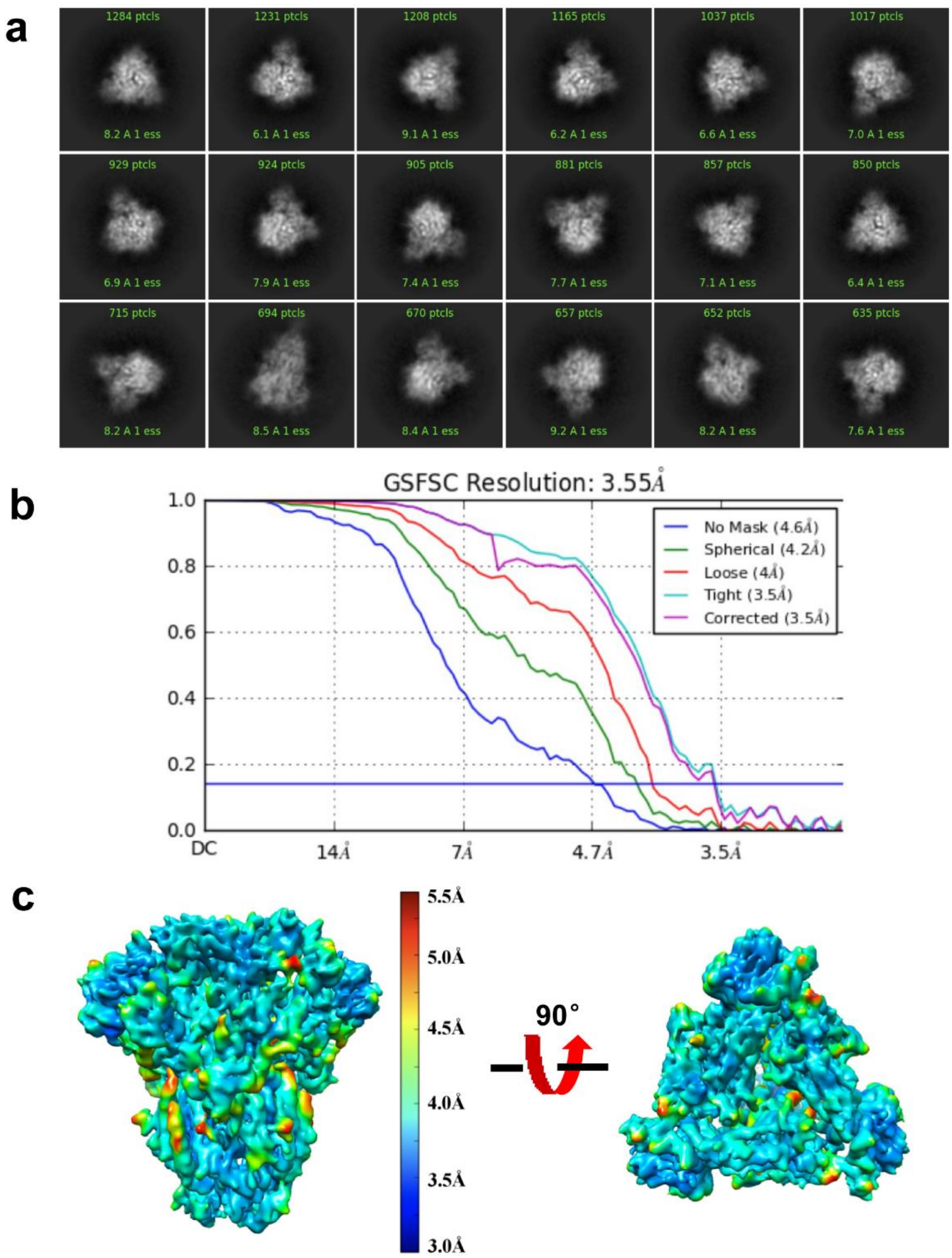

Supplementary Figure 4. Cryo-EM analysis of the conformation 2.

(a) Representative 2D class averages of the conformation 2 used in the final refinement.

(b) Gold-standard Fourier shell correlation (FSC) curves of conformation 2. The 0.143 cut-off value is indicated by a horizontal cyan line. (c) Local resolution map of conformation 2 density map analyzed by the local resolution estimation tool in cryoSPARC.

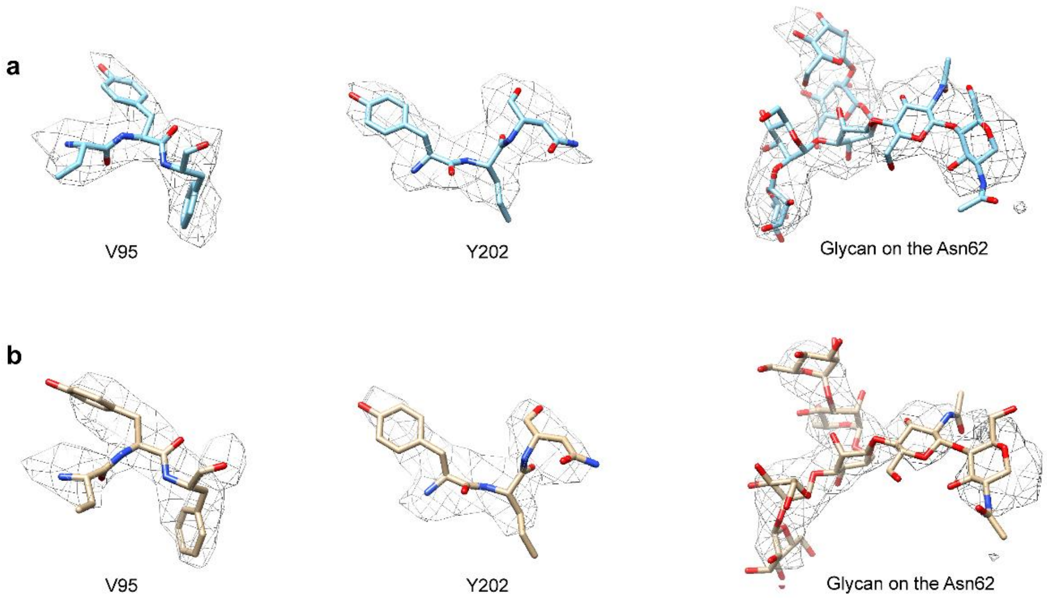

Supplementary Figure 5. The representative density maps of HCoV-229E C1 (a) and C2 (b).

The residues (Val95, Tyr202) and glycan on the Asn62 are fitted into the corresponding maps. The map is contoured at 2.0 RMS to show the density.

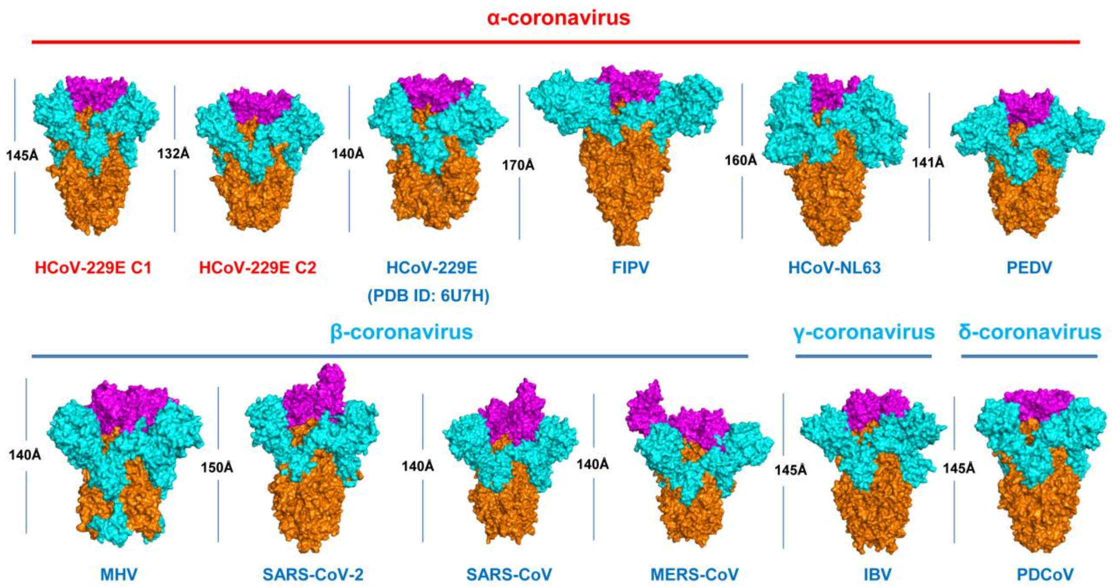

Supplementary Figure 6. The overall structure comparison of coronavirus S trimers.

The S trimer structures of HCoV-229E (C1, C2 and PDB ID: 6U7H), HCoV-NL63

(PDB ID: 5SZS), PEDV (PDB ID: 6U7K), FIPV (PDB ID: 6JX7), MHV (PDB ID: 3JCL), SARS-CoV-2 (PDB ID: 6VSB), SARS-CoV (PDB ID: 5X5B), MERS-CoV (PDB ID: 5X5F), IBV (PDB ID: 6CV0), and PDCoV (PDB ID: 6BFU) are shown. The S1-RBDs and S2 subunit are colored as magenta and orange, respectively. The lengths of conformations 1 and 2 are measured via the PyMOL. The lengths of the other structures are shown as previous reports.

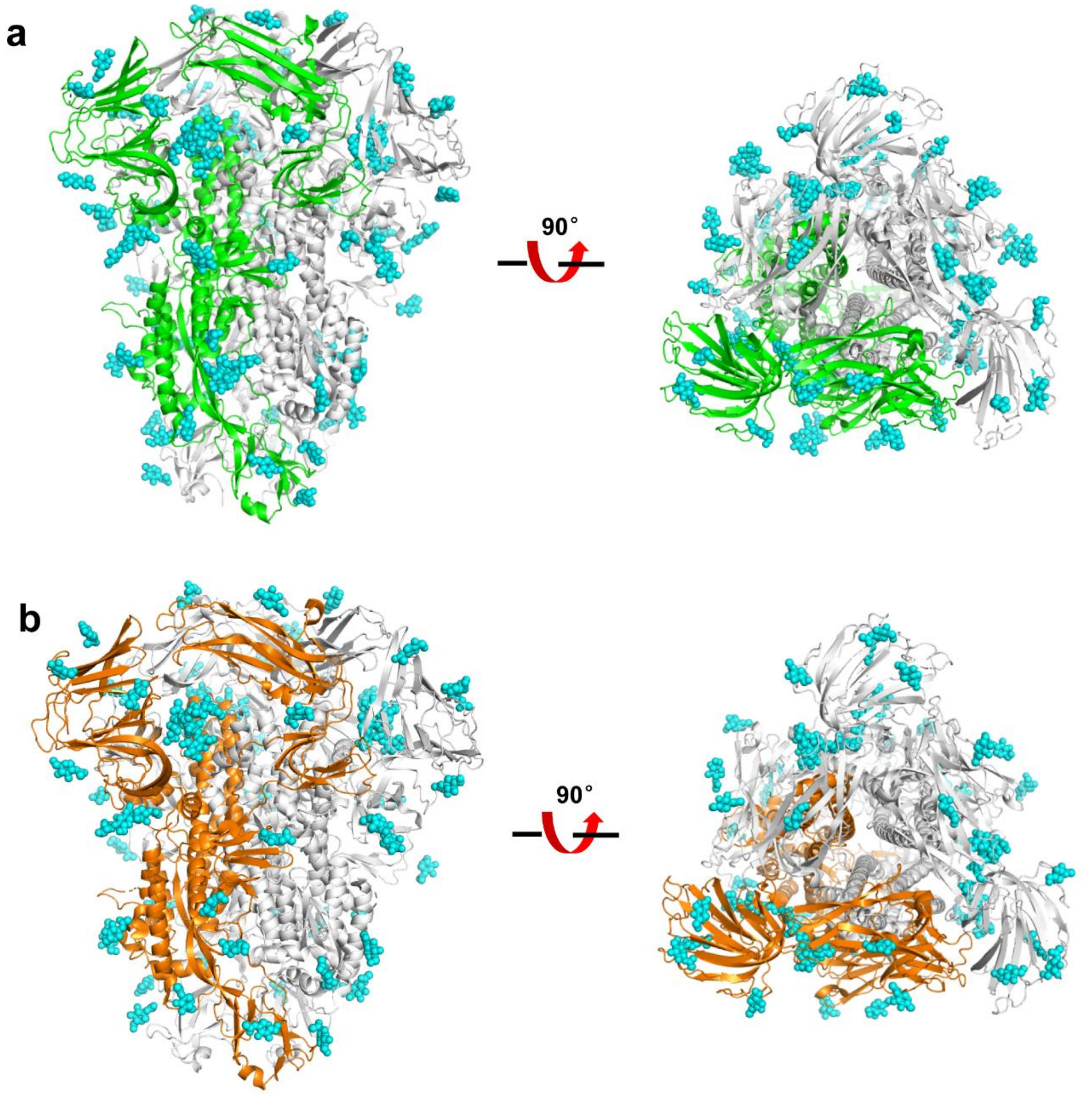

Supplementary Figure 7. Glycan sites of HCoV-229E C1 (a) and C2 (b).

Glycans are colored in cyan. The chain A of C1 and C2 is colored in green and orange,

respectively. The others are colored in gray.

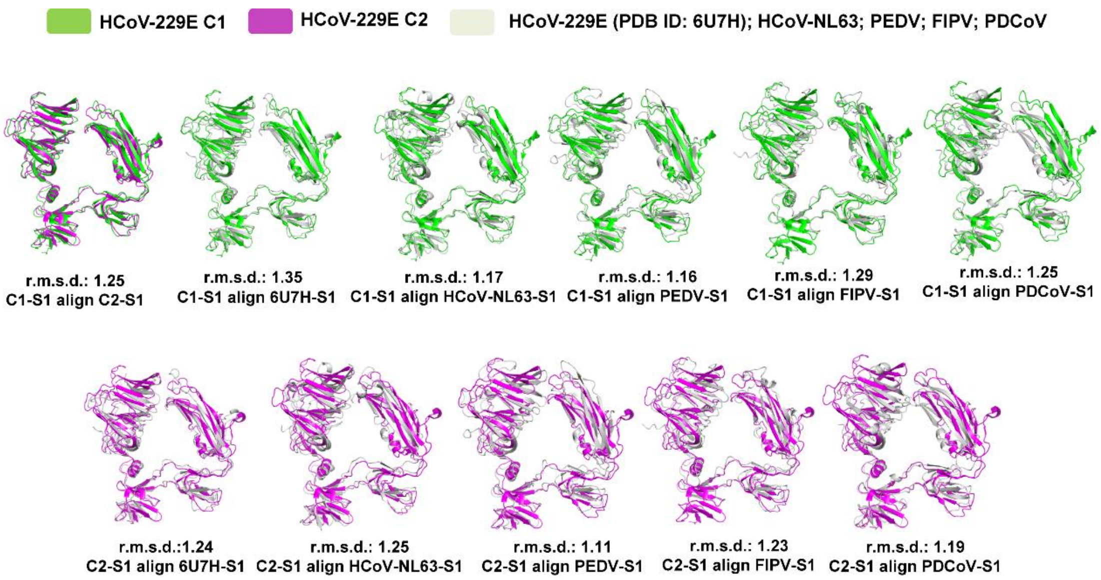

Supplementary Figure 8. Structural comparison of coronaviruses S1 subunits.

The S1 subunits of HCoV-229E (C1, C2 and PDB ID: 6U7H), HCoV-NL63, PEDV, FIPV and PDCoV are shown. The S1 subunits of C1 and C2 are colored in green and magenta, respectively. Besides, the S1 subunits of other coronaviruses (HCoV-229E with PDB ID: 6U7H, HCoV-NL63, PEDV, FIPV and PDCoV) are colored in gray. The r.m.s.d. values of the Cα atoms are also shown. The PDB IDs are the same as those in Supplementary Figure 6.

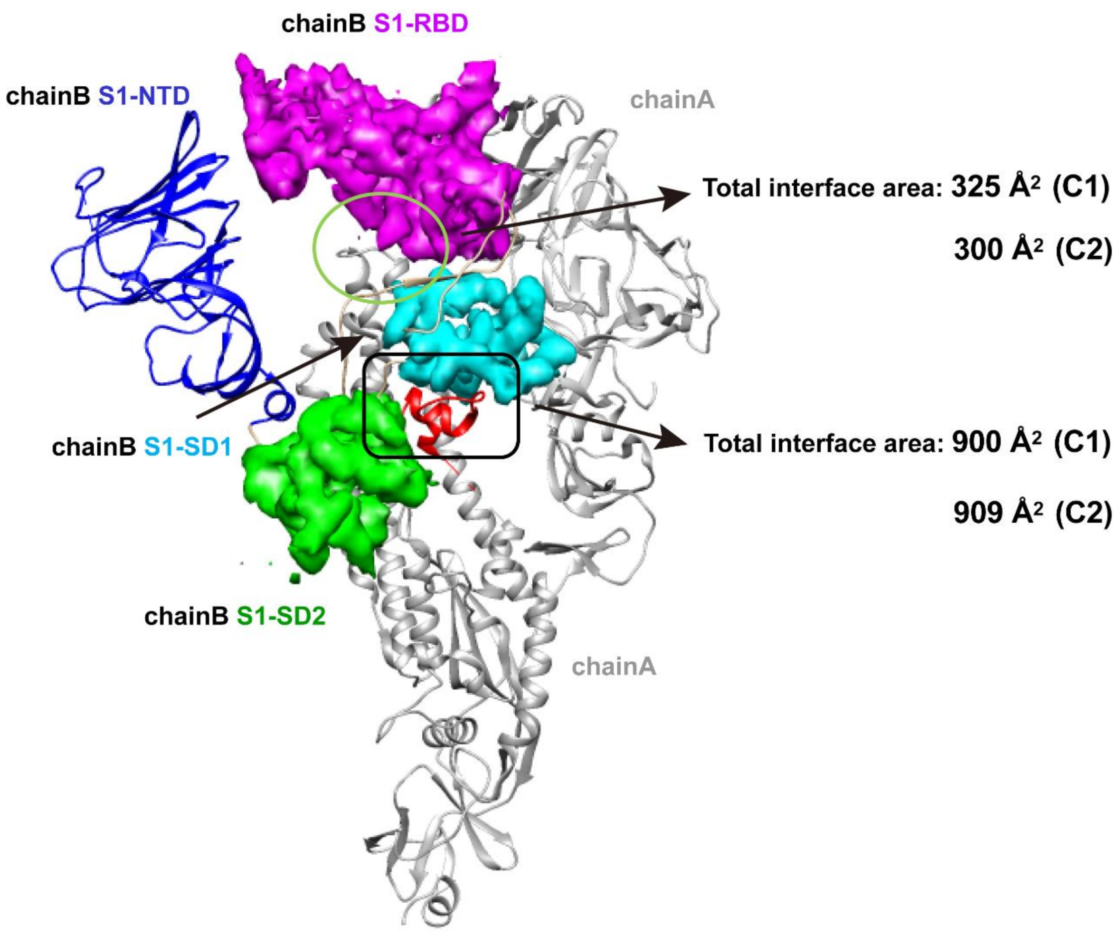

Supplementary Figure 9. The S1 subunits stabilize S2 subunits with hydrophobic interations.

The hydrophobic interface area between the RBD (chain B) and surrounding CH-HR1 junction (chain A, Ile865-Ala874) were around 325 Å^2 (C1) and 300 Å^2 (C2), shown in green ellipse. The SD1 and SD2 domains of each S1 subunit clamp over a hydrophobic knob (residues Ala709-pro737) that protrudes from the helical core region of each S2 subunit, resulting in around 900 Å^2 (C1) and 909 Å^2 (C2) of interface area (with a total of ~2700 Å^2 and ~2727 Å^2, respectively). The interface areas are analyzed using PDBePISA. The chain C and the S2 subunit of chain B are omitted for clarification.

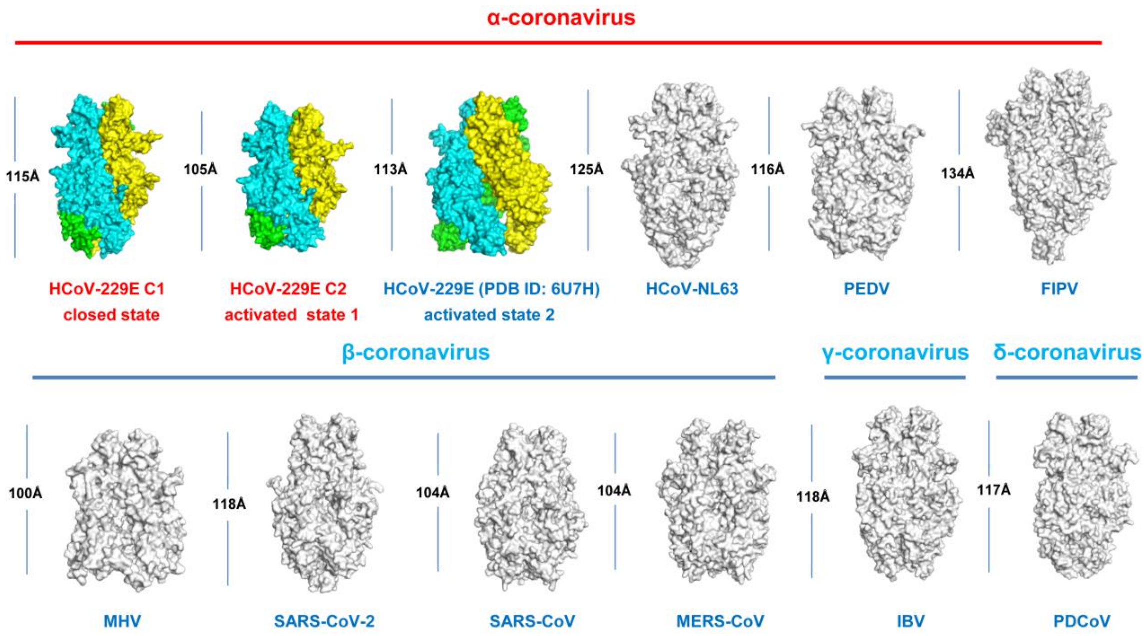

Supplementary Figure 10. Structural comparison of coronavirus S2 trimers.

The S2 trimer structures of HCoV-229E (C1, C2 and PDB ID: 6U7H), HCoV-NL63, PEDV, FIPV, MHV, SARS-CoV-2, SARS-CoV, MERS-CoV, IBV and PDCoV are shown. Chains A, B and C of C1, C2 and HCoV-229E (PDB ID: 6U7H) are colored in cyan, yellow and green, respectively. The S2 trimers of other coronaviruses (HCoV-NL63, PEDV, FIPV, MHV, HCoV-HKU1, SARS-CoV, MERS-CoV, IBV and PDCoV) are colored in gray. The PDB IDs are the same as those in Supplementary Figure 6.

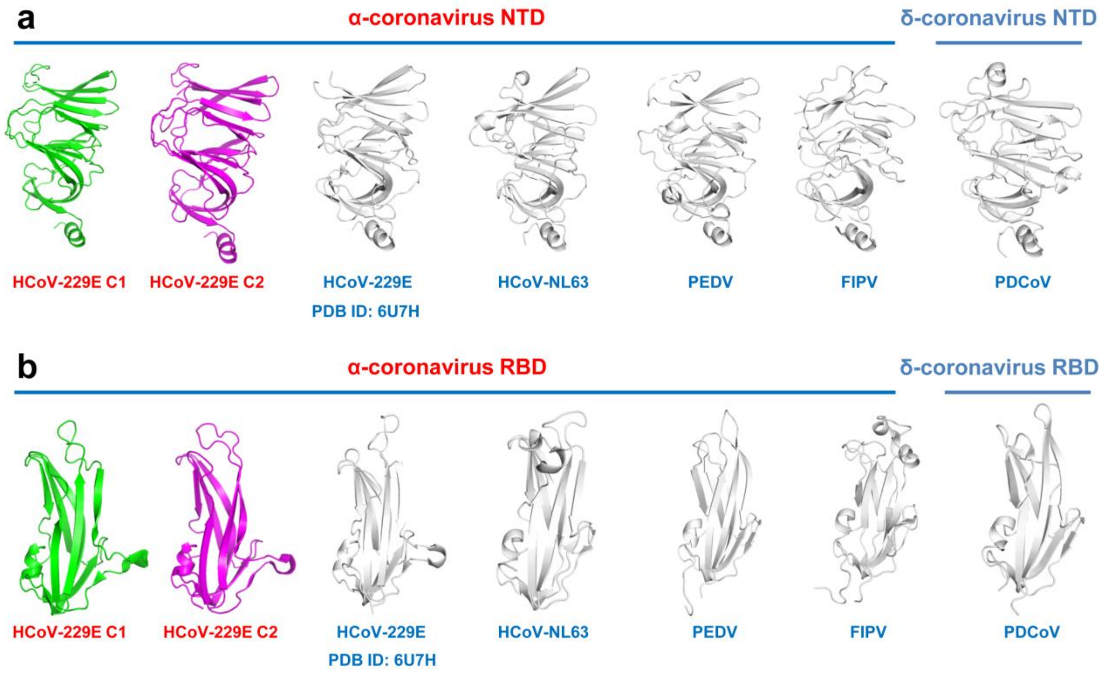

Supplementary Figure 11. Structural comparison of coronavirus S1-NTDs and S1-RBDs.

The S1-NTDs and S1-RBDs from HCoV-229E (C1, C2 and PDB ID: 6U7H), HCoV-NL63, PEDV, FIPV and PDCoV are shown. The NTDs and RBDs of C1 and C2 are colored in green and magenta, respectively. Other coronaviruses (HCoV-229E with PDB ID: 6U7H, HCoV-NL63, PEDV, FIPV and PDCoV) S1-NTDs and S1-RBDs are colored in gray. The PDB IDs are the same as those in Supplementary Figure 6.

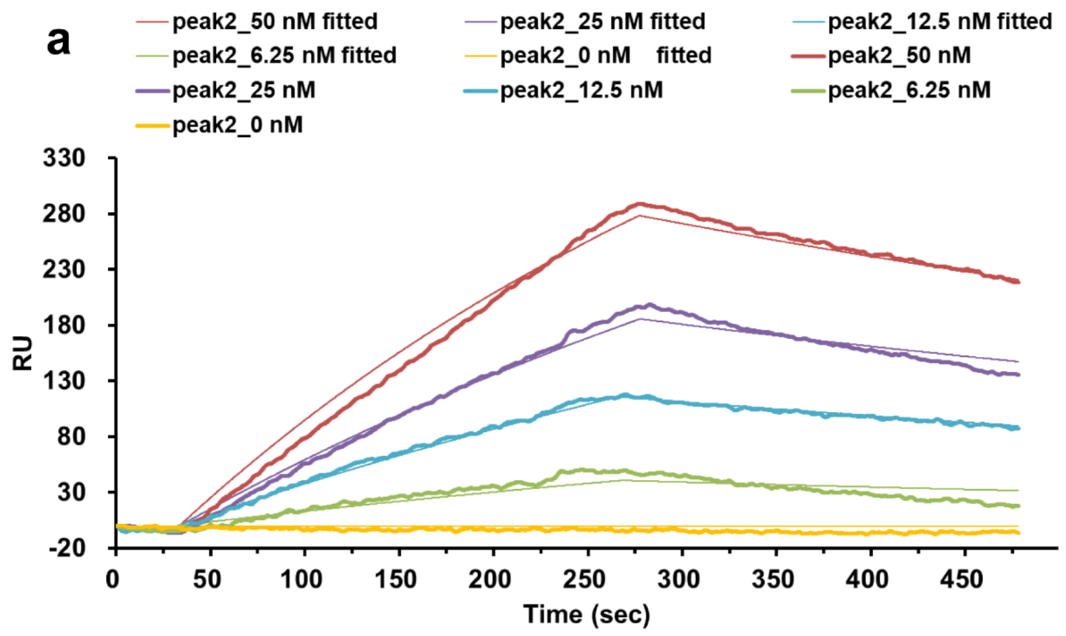

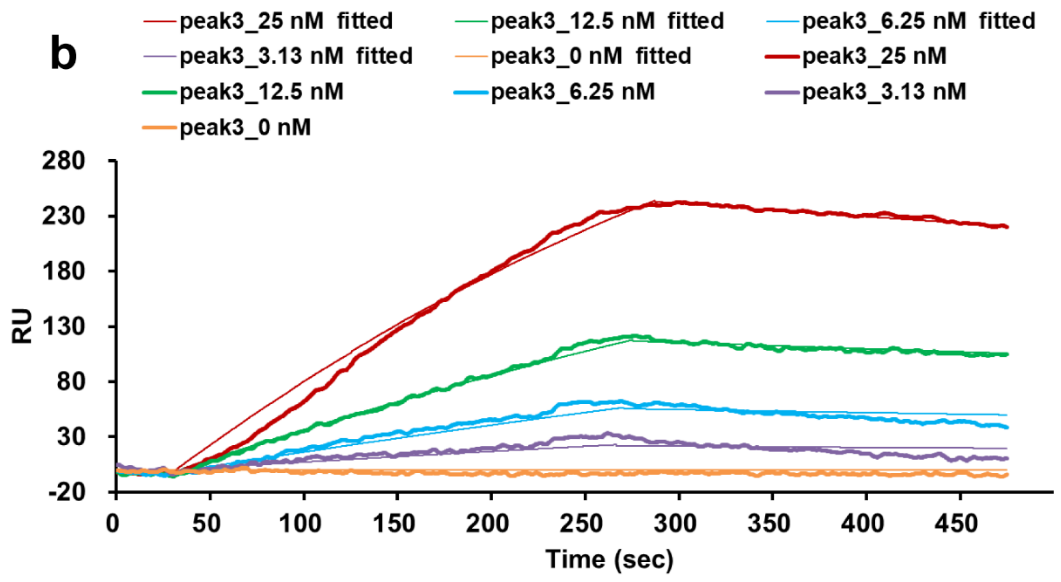

Supplementary Figure 12. Interactions of HCoV-229E S proteins (peak 2 and 3 sample) and soluble hAPN.

The SPR sensorgrams of peak2 sample (a) and peak3 sample (b) displaying the binding beween the S proteins and soluble hAPN receptors are shown. Data are shown as different coloured lines and binding kinetics were evaluated using a 1:1 Langmuir binding model. The experiments were repeated two times independently with similar results, and one representative set of curves is shown.

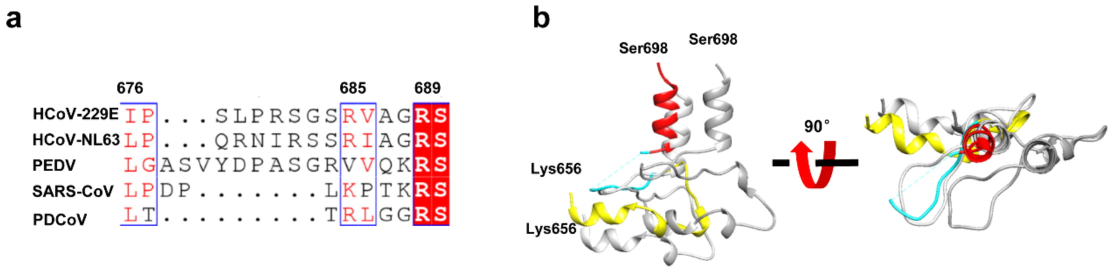

Supplementary Figure 13. Structural and sequence analysis of the HCoV-229E S2′ trigger loop.

(a) Sequence alignment of the S2′ trigger loop from HCoV-229E (GenBank ID: NP_073551.1), HCoV-NL63 (GenBank ID: UniProt Q6Q1S2), PEDV (GenBank ID: KC140102.1), SARS-CoV (GenBank ID: NP_828851.1) and PDCoV (GenBank ID: KT336560). (b) Structural alignment of the S2′ trigger loop of C1 (gray), C2 (colorful) and the previous reported open S protein (PDB ID: 6U7H, dark gray).

Supplementary Table 1 | Data collection and refinement statistics

**Conformations**	1 (EMD-9744) (6IXA)	2 (EMD-9745) (6IXB)
**Data collection and processing**		
Magnification	18, 000	18, 000
Voltage (kV)	300	300
Electron exposure (e^{-} Å^{-2})	60	60
Defocus range (μm)	2-3	2-3
Pixel size (Å)	1.40	1.40
Symmetry imposed	C3	C3
images (no.)	2779	5081
particles (no.)	403,347	659,410
Map resolution (Å)	3.21	3.55
FSC threshold	0.143	0.143
**Model statistics**		
Model vs. Map CC (volume)	0.78	0.77
MolProbity Score	2.81	3.10
**Ramachandran statistics**		
Most favored	93.90	94.34
Allowed	5.57	5.56
Outliers	0.53	0.10
**RMS deviations**		
Bond lengths(Å)	0.007	0.008
Bond angles(°)	1.142	1.164
