# Supplementary material for: Cryo-EM analysis of the HCoV-229E spike glycoprotein reveals dynamic prefusion conformational changes
Source: Nat Commun. 2021 Jan 8;12:141. doi: 10.1038/s41467-020-20401-y (PMC7794242; doi:10.1038/s41467-020-20401-y)
Supplement: Supplementary file 3 — Reporting Summary [file 41467_2020_20401_MOESM3_ESM.pdf]

nature research

Corresponding author(s): Guiqing Peng and Zhi-jie Liu

Last updated by author(s): Sep 15, 2020

Reporting Summary

Nature Research wishes to improve the reproducibility of the work that we publish. This form provides structure for consistency and transparency in reporting. For further information on Nature Research policies, see our Editorial Policies and the Editorial Policy Checklist.

Statistics

For all statistical analyses, confirm that the following items are present in the figure legend, table legend, main text, or Methods section.

n/a	Confirmed	
☐	☒	The exact sample size (*n*) for each experimental group/condition, given as a discrete number and unit of measurement
☐	☒	A statement on whether measurements were taken from distinct samples or whether the same sample was measured repeatedly
☐	☒	The statistical test(s) used AND whether they are one- or two-sided<br>*Only common tests should be described solely by name; describe more complex techniques in the Methods section.*
☒	☐	A description of all covariates tested
☒	☐	A description of any assumptions or corrections, such as tests of normality and adjustment for multiple comparisons
☐	☒	A full description of the statistical parameters including central tendency (e.g. means) or other basic estimates (e.g. regression coefficient) AND variation (e.g. standard deviation) or associated estimates of uncertainty (e.g. confidence intervals)
☐	☒	For null hypothesis testing, the test statistic (e.g. *F*, *t*, *r*) with confidence intervals, effect sizes, degrees of freedom and *P* value noted<br>*Give P values as exact values whenever suitable.*
☒	☐	For Bayesian analysis, information on the choice of priors and Markov chain Monte Carlo settings
☒	☐	For hierarchical and complex designs, identification of the appropriate level for tests and full reporting of outcomes
☒	☐	Estimates of effect sizes (e.g. Cohen's *d*, Pearson's *r*), indicating how they were calculated

Our web collection on statistics for biologists contains articles on many of the points above.

Software and code

Policy information about availability of computer code

Data collection	SerialEM software (Version 3.6.1)
Data analysis	CryoSPARC (Version 2.14.2), COOT (Version 0.9.1) and PHENIX (Version 1.18-3855-000) were used at the determination of the structure for data processing , model building and refinement. PyMOL (Version 2.4.0) and Chimera (Version 1.14) were used to generate the structural figures. Statistical analysis was done using GraphPad Prism 7.0 (GraphPad Software). Binding kinetic parameters were obtained by fitting the curve to a one-to-one binding model using the TraceDrawer software package (Version 1.8).

For manuscripts utilizing custom algorithms or software that are central to the research but not yet described in published literature, software must be made available to editors and reviewers. We strongly encourage code deposition in a community repository (e.g. GitHub). See the Nature Research guidelines for submitting code & software for further information.

Data

Policy information about availability of data

All manuscripts must include a data availability statement. This statement should provide the following information, where applicable:

- Accession codes, unique identifiers, or web links for publicly available datasets
- A list of figures that have associated raw data
- A description of any restrictions on data availability

EM maps have been deposited in EMDB with accession codes EMD-9744 (conformation 1) and EMD-9745 (conformation 2). In addition, the modeled atomic coordinates have been deposited in the Protein Data Bank with the accession code 6IXA (conformation 1) and 6IXB (conformation 2). Other data are available from the corresponding author upon reasonable request.

Field-specific reporting

Please select the one below that is the best fit for your research. If you are not sure, read the appropriate sections before making your selection.

☒ Life sciences ☐ Behavioural & social sciences ☐ Ecological, evolutionary & environmental sciences

For a reference copy of the document with all sections, see nature.com/documents/nr-reporting-summary-flat.pdf

Life sciences study design

All studies must disclose on these points even when the disclosure is negative.

Sample size	Sample size choice was based on previous studies (ref.- 11, 26). Statistical analysis was carried out using GraphPad Prism 7.0. Statistical analysis was conducted on data from three independent experimental replicates. Statistical significance was determined using an unpaired two-tailed Student's t test. Data are presented as mean values +/- SD (95% confidence interval). All differences between means with p < 0.05 are indicated. *p<0.05, **p<0.01, and ***p<0.001 were considered significant.
Data exclusions	There are no data excluded from the analyses.
Replication	Sample size choice was based on previous studies (ref.- 11, 26). Pseudotyped-virus entry assay was repeated 3 times and the result is determined based on the reproducibility of the experiments. SPR assays were repeated independently twice with similar results. All attempts at replication were successful.<br>Statistical analysis was carried out using GraphPad Prism 7.0. Statistical analysis was conducted on data from three independent experimental replicates. Statistical significance was determined using an unpaired two-tailed Student's t test. Data are presented as mean values +/- SD (95% confidence interval). All differences between means with p < 0.05 are indicated. *p<0.05, **p<0.01, and ***p<0.001 were considered significant.
Randomization	No randomization method was used. Because randomization is not relevant to this study.
Blinding	Data collection and Analysis was not performed blind. Because blinding has no effect on the experiment results.

Reporting for specific materials, systems and methods

We require information from authors about some types of materials, experimental systems and methods used in many studies. Here, indicate whether each material, system or method listed is relevant to your study. If you are not sure if a list item applies to your research, read the appropriate section before selecting a response.

Materials & experimental systems

n/a	Involved in the study	
☒	☐	Antibodies
☐	☒	Eukaryotic cell lines
☒	☐	Palaeontology and archaeology
☒	☐	Animals and other organisms
☒	☐	Human research participants
☒	☐	Clinical data
☒	☐	Dual use research of concern

Methods

n/a	Involved in the study	
☒	☐	ChIP-seq
☒	☐	Flow cytometry
☒	☐	MRI-based neuroimaging

Eukaryotic cell lines

Policy information about cell lines

Cell line source(s)	Sf9, HEK293T and Huh7 are from ATCC.
Authentication	Cell line was directly purchased from Distributors. Cell line authentication was not performed.
Mycoplasma contamination	Sf9, HEK293T and Huh7 were negative for mycoplasma contamination.
Commonly misidentified lines (See ICLAC register)	No commonly misidentified cell lines were used.
