# Supplementary material for: Cryo-EM analysis of the HCoV-229E spike glycoprotein reveals dynamic prefusion conformational changes
Source: Nat Commun. 2021 Jan 8;12:141. doi: 10.1038/s41467-020-20401-y (PMC7794242; doi:10.1038/s41467-020-20401-y)
Supplement: Supplementary file 4 — Description of Additional Supplementary Files [file 41467_2020_20401_MOESM4_ESM.pdf]

Description of Additional Supplementary Files

File Name: Supplementary Movie 1

Description: Conformation morphing from C1 (gray) to C2 (hot pink).

File Name: Supplementary Movie 2

Description: Conformation morphing from C2 (hot pink) to open state (dim gray).

File Name: Supplementary Movie 3

Description: Conformation morphing of HCoV-229E spike trimer.
